# Enhanced Antibacterial and Immunomodulatory Porphyrin-Based MOF Coatings for PETG Clear Aligners: A Comparative Study of Ag, Cu, and Ce Metal Centers

**DOI:** 10.3390/ijms27125411

**Published:** 2026-06-16

**Authors:** Zhaoping Sang, Bowen Tang, Yunhao Zhuo, Lixin Li, Qi Zhang, Yinan Jin, Huiming Zhang, Gang Zhao

**Affiliations:** 1 School of Stomatology, Jiamusi University, Jiamusi 154000, China; 15145407770@163.com (Z.S.); 15140107212@163.com (B.T.); z137048110@163.com (Y.Z.); 13359522591@163.com (L.L.); 18003645977@163.com (Q.Z.); 18746363690@163.com (Y.J.); 2 College of Basic Medical Sciences, Jiamusi University, Jiamusi 154000, China

**Keywords:** MOF, clear aligners, antibacterial performance, coating, anti-biofilm

## Abstract

Prolonged use of clear aligners promotes bacterial colonization and biofilm formation, which can compromise orthodontic outcomes. There is a clear clinical demand for approaches that can suppress pathogenic activity while preserving the fundamental functional and material characteristics of the aligners. To address this need, a novel strategy of fabricating metal–organic framework (MOF) coatings on aligners was adopted. Metal–organic frameworks (MOFs) have emerged as promising antibacterial coating materials by combining antimicrobial metal ions with biocompatible organic ligands. Three distinct porphyrin-based MOFs (Ag-, Cu-, and Ce-TCPP) were synthesized and fabricated as coatings on clear aligner surfaces via a coordination-driven self-assembly approach. The coated aligners were comprehensively assessed in vitro to determine their antibacterial performance, anti-inflammatory potential, biocompatibility, and key physical characteristics. Among the three coatings, Ag-TCPP showed the most favorable overall antibacterial and anti-biofilm performance in the present experimental system and facilitated macrophage polarization toward an anti-inflammatory M2-like phenotype. Ag-TCPP exhibited a significant inhibition zone of 6.75 ± 0.25 mm and reduced biofilm biomass by 72.2%. All MOF coatings exhibited excellent biocompatibility, and their application did not compromise the aligners’ mechanical integrity or aesthetic properties (light transmittance). This study reports the successful development of a novel metal–organic framework (MOF)-based coating strategy for clear aligners. Among the formulations investigated, the Ag-TCPP coating exhibited outstanding antibacterial and immunomodulatory performance while maintaining the critical mechanical integrity and aesthetic qualities of the aligner. The findings of this work offer a practical approach to designing multifunctional orthodontic devices that may reduce biofilm-related complications and improve clinical outcomes.

## 1. Introduction

The pursuit of aesthetic perfection, driven by rising living standards and the increasing emphasis on dental health and aesthetics, has contributed to the increasing preference for clear aligners in orthodontic treatment. While clear aligners are associated with reduced plaque accumulation compared to fixed appliances, empirical evidence indicates that there is no significant improvement in oral microbial composition [1] or a meaningful reduction in bacterial load [2] during treatment. Thus, inadequate oral hygiene following orthodontic treatment results in complications such as tooth demineralization and periodontal inflammation [3]. Existing clinical approaches, such as mechanical debridement and site-specific antibiotic administration, are limited by several challenges, including substantial financial burden, frequent disease recurrence, and the potential interference with periodontal tissue healing and regeneration. Antibiotic-based therapies are also constrained by restricted antimicrobial spectra, the growing prevalence of antimicrobial resistance, and reduced effectiveness against pathogens embedded within biofilm structures [4,5]. There is thus an urgent need for non-surgical, antibiotic-free strategies for the effective elimination of oral pathogens. Modification of aligner materials to inhibit bacterial proliferation represents a promising approach; modifications that induce changes in the mechanical properties of thermoplastic materials may adversely affect the efficiency of tooth movement. Surface coatings offer a viable solution by introducing additional functionalities without compromising mechanical performance [6]. Despite their apparent benefits, research on coatings for clear aligners remains limited and calls for further in-depth investigation.

Antibacterial metal–organic framework (MOF) coatings, integrating antimicrobial metal ions and biocompatible organic ligands, have emerged as a promising biomedical coating material. The antimicrobial activity is mediated via the adsorption of metal ions onto bacterial surfaces, leading to membrane disruption, impairment of cellular metabolism, and pathogen inactivation. Moreover, specific organic ligands facilitate the generation of reactive oxygen species (ROS) (e.g., singlet oxygen, superoxide radicals, and hydrogen peroxide), which mediate microbial sterilization through redox-driven mechanisms [7]. MOFs’ high surface area, porosity, and nano-architecture may also enable the controlled storage and release of metal ions in response to specific environmental stimuli [8]. Variations in MOF structures influence ion release kinetics and antimicrobial efficacy, reflecting their tunable properties. Porphyrinic ligands such as TCPP were selected in this study because of their unique multifunctional structure. Compared with simple dicarboxylate ligands such as terephthalic acid, TCPP contains four peripheral carboxylate groups, an extended π-conjugated macrocycle, and a central coordination cavity, which facilitate coordination with metal centers and the formation of stable porphyrin-based MOF structures [9]. Although TCPP is more costly than some commonly used organic linkers, its structural multifunctionality makes it suitable for constructing antibacterial and immunomodulatory MOF coatings.

To the best of our knowledge, this is the first report on the functionalization of clear aligners using MOF-based coatings. By employing a coordination-driven self-assembly approach, three distinct MOF coatings (Ag-TCPP, Cu-TCPP, and Ce-TCPP) were successfully fabricated on the aligner surface. The polymer substrate surface was selectively dissolved using a novel organic chemical method, significantly enhancing the binding strength and stability of the MOF coatings. In vitro studies demonstrated that the developed coatings demonstrated excellent biocompatibility, antibacterial efficacy, and anti-inflammatory performance while preserving the mechanical and aesthetic properties of the aligners. This study advances our understanding of bioactive surface modifications as well as the functional application of MOFs in orthodontic materials. It also establishes a novel framework for the development of antibacterial coatings for clear aligners.

## 2. Results

### 2.1. Characterization of MOF-Coated Aligners

**Figure 1:**
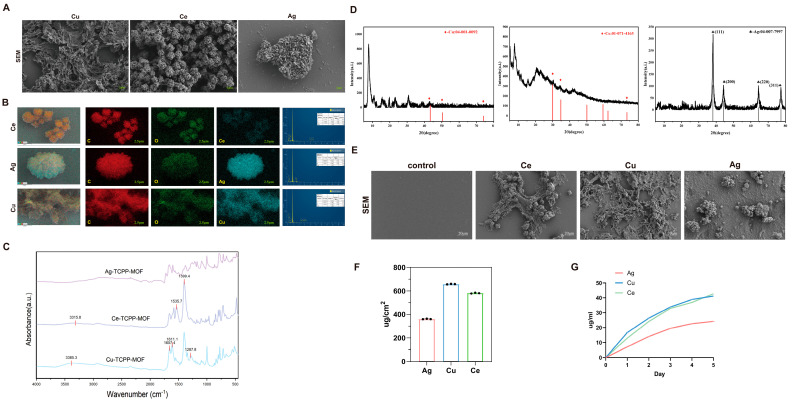
Synthesis and Characterization: (**A**) SEM images at 5000× magnification showing the morphology of synthesized Cu-, Ce-, and Ag-TCPP MOFs. (**B**) EDS analysis showing the surface elemental composition of MOF-coated aligners. (**C**) Fourier-transform infrared (FTIR) spectra of Ag-, Ce-, and Cu-TCPP MOFs. (**D**) The X-ray diffraction (XRD) patterns of Ag-, Ce-, and Cu-TCPP MOFs. (**E**). SEM images of uncoated and three MOF-coated aligners. (**F**) Initial surface metal ion concentration on three aligners prepared with a 300 μg/mL MOF coating. (**G**) Sustained release profiles of metal ions from three types of MOF-coated aligners in artificial saliva over 5 days.

Microstructural characteristics of Ag-TCPP, Cu-TCPP, and Ce-TCPP metal–organic frameworks (MOFs) were analyzed using scanning electron microscopy (SEM, Figure 1A). Cu-TCPP MOFs exhibited a characteristic flocculent morphology, comprising interwoven nanofibers that developed into a well-organized three-dimensional porous network with consistent morphology. Ce-TCPP MOFs, on the other hand, presented well-defined block-like structures with distinct edges and smooth surfaces. Ag-TCPP MOFs heterogeneously existed as discrete block-like particles but with significantly larger dimensions and without interparticle connectivity. Energy-dispersive X-ray spectroscopy (EDS) confirmed the elemental composition of the MOF-coated aligner surfaces, confirming the existence of carbon, oxygen, and respective metal elements (Figure 1B). To improve readability, enlarged FT-IR spectra and PXRD patterns are provided in Supplementary Figure S1, allowing clearer visualization of the characteristic absorption bands and diffraction peaks.

Fourier-transform infrared (FTIR) spectra showed weak –OH stretching peaks at 3315.8 cm^−1^ (Ce-TCPP MOFs) and 3385.3 cm^−1^ (Cu-TCPP MOFs). The corresponding band was, however, absent in Ag-TCPP MOFs, indicating differential –OH coordination involvement. The structural framework for Ag-TCPP MOFs was predominantly stabilized by Ag–N bonds (Figure 1C). X-ray diffraction (XRD) analysis (Figure 1D) revealed distinct diffraction patterns for each MOF variant, due to the difference in their metal counterparts. X-ray diffraction (XRD) analysis revealed distinct diffraction patterns among the TCPP-based metal-containing materials. Ce-TCPP and Cu-TCPP displayed characteristic peaks at 7.8°, 10.9°, 12.2°, and 19.6°, which were assigned to the (100), (002), (210), and (004) planes, respectively. For Ag-TCPP, the peaks at approximately 38.2°, 44.2°, 64.4°, and 77.4° corresponded to the (111), (200), (220), and (311) planes of metallic Ag, respectively. These findings indicate the presence of metallic Ag secondary phases in the Ag-TCPP sample. Therefore, Ag-TCPP is more appropriately described as an Ag–TCPP coordination/MOF-based composite containing metallic Ag, rather than a single-phase pure MOF material.

These results suggest that partial reduction of Ag^+^ occurred during synthesis and/or post-treatment, resulting in metallic Ag secondary phases. Therefore, Ag-TCPP should be more cautiously regarded as an Ag–TCPP coordination/MOF-based composite containing metallic Ag secondary phases, rather than a single-phase pure MOF material. Although the current FT-IR and PXRD results support the formation of metal–TCPP coordination structures, they are not sufficient to fully determine the detailed crystallographic structures or phase purity of all samples. Further characterization, including PXRD comparison with the free TCPP ligand, comparison with simulated or previously reported PXRD patterns, and additional spectroscopic analysis, is still required to unambiguously confirm the detailed structures.

Surface morphological analysis (Figure 1E) demonstrated that the MOF-coated aligners retained the structural features of the pristine MOF nanoparticles in solution, confirming their successful deposition on the aligner surfaces.

### 2.2. Metal Ion Sustained-Release Testing of Aligner Coatings

Surface metal ion concentrations on the MOF-coated aligners were quantitatively determined by inductively coupled plasma mass spectrometry (ICP-MS) analysis (Figure 1F), yielding values of 658 μg/cm^2^ for the Cu-based group, 360 μg/cm^2^ for the Ag-based group, and 581 μg/cm^2^ for the Ce-based group. The MOF-coated aligners exhibited sustained metal ion release in artificial saliva over 1–5 days (Figure 1G), suggesting that metal ion release may contribute to their antibacterial activity under simulated oral conditions.

### 2.3. Mechanical and Optical Characterization of MOF-Coated Aligners

**Figure 2:**
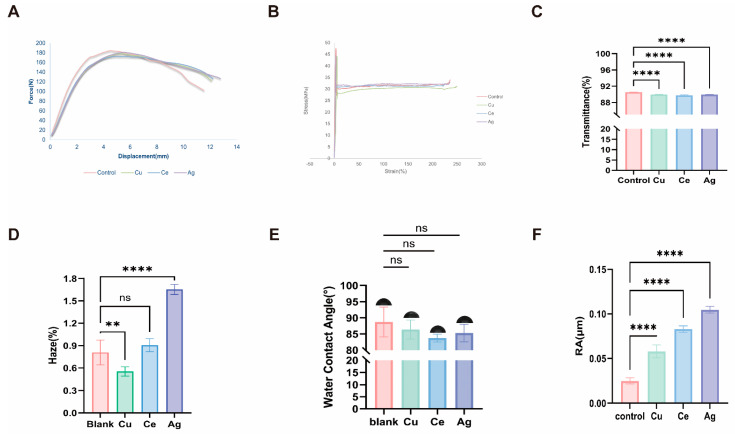
Mechanical and optical properties: (**A**) Right-angle tear strength of uncoated and Cu-, Ag-, and Ce-TCPP MOF-coated aligners. (**B**) Stress-stain curves of uncoated and three TCPP MOF-coated aligners under tensile loading. (**C**) Light transmittance (%) of uncoated and Cu-, Ag-, and Ce-TCPP MOF-coated aligners. (**D**) Haze (%) of uncoated and three TCPP MOF-coated aligners. (**E**) Water contact angle (WCA, °) of uncoated and Cu-TCPP, Ag-TCPP, and Ce-TCPP MOF-coated aligners. (**F**) Arithmetic Roughness Average (Ra, μm) of uncoated and three TCPP MOF-coated aligners. **: *p* < 0.01, ****: *p* < 0.0001, ns: no significance.

Mechanical performance evaluation through stress–strain analysis and right-angle tear strength measurements (Figure 2A,B) indicated that Biolon aligners retained their mechanical performance following MOF surface modification, preserving their clinical efficacy during the application of mechanical force in orthodontic therapy. Optical transparency evaluation (Figure 2C) showed no significant changes in light transmittance across treatment groups. While Ag-TCPP-coated aligners exhibited a marginal increase in haze, induced by Ag-derived particulate species, both Cu-TCPP and Ce-TCPP aligners preserved optical performance equivalent to that of the uncoated samples (Figure 2D). Surface analysis demonstrated no significant differences in water contact angles (*p* > 0.05) between coated and uncoated aligners (Figure 2E). Surface profilometric evaluation showed a significant increase in roughness exclusively in the Ag-TCPP-coated aligners (Figure 2F).

### 2.4. Antibacterial Evaluation of TCPP-Based MOFs

**Figure 3:**
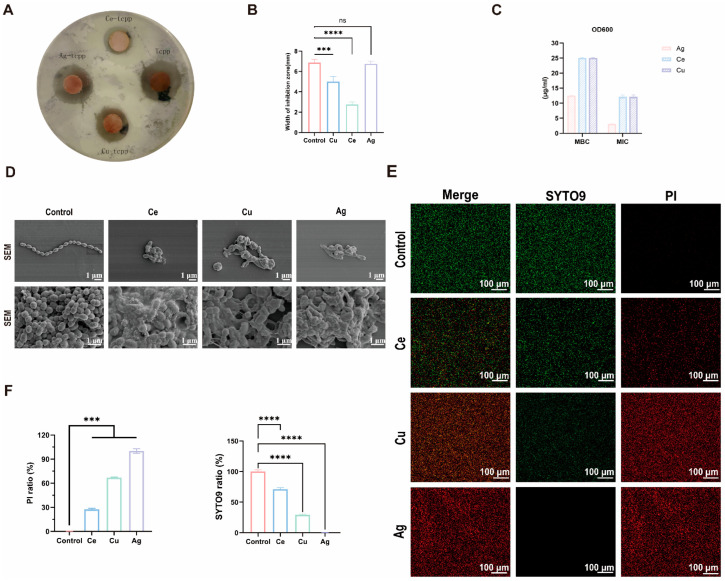
Broad-spectrum Antibacterial Activity: (**A**) Zone of inhibition (ZOI) of the three TCPP MOFs and TCPP against *Streptococcus mutans*. (**B**) Quantitative analysis of the width of inhibition zones (ZOI) against bacteria. (**C**) Minimum inhibitory concentration (MIC) and minimum bactericidal concentration (MBC) of Cu-, Ag-, and Ce-TCPP MOFs. (**D**) Scanning electron microscopy (SEM) images showing morphological changes in bacteria treated with MOF-coated aligner extracts. (**E**) Confocal laser scanning microscopy (CLSM) images of Live/Dead staining of *Streptococcus mutans* (*S. mutans*) exposed to Cu-TCPP MOFs, Ag-TCPP MOFs, and Ce-TCPP MOFs. (**F**) Quantitative analysis of Live/Dead staining ratios (viable vs. non-viable cells) for the three TCPP MOFs. ***: *p* < 0.001, ****: *p* < 0.0001, ns indicates no significant difference (*p* ≥ 5).

The antibacterial efficacy of three TCPP-based metal–organic frameworks (MOFs) was systematically evaluated through multiple assays. As shown in Figure 3A,B, zone of inhibition (ZOI) analyses revealed distinct antibacterial performance across the tested materials, with Ag-TCPP MOFs exhibiting significantly larger inhibition zones compared to both Cu-TCPP and Ce-TCPP MOFs, which demonstrated a reduced activity relative to free TCPP. Quantitative evaluation determined that the minimum inhibitory concentrations (MICs) were 3.13 μg/mL for Ag-TCPP and 12.50 μg/mL for both Cu-TCPP and Ce-TCPP, with the corresponding minimum bactericidal concentrations (MBCs) exhibiting a comparable pattern (Figure 3C). The inhibition zone widths were 6.87 ± 0.32 mm, 5.00 ± 0.50 mm, 2.75 ± 0.25 mm, and 6.75 ± 0.25 mm for the TCPP, Cu, Ce, and Ag groups, respectively. Compared with the TCPP group, the Cu and Ce groups showed significantly narrower inhibition zones (*p* < 0.001 and *p* < 0.0001, respectively), whereas no significant difference was observed between the Ag and TCPP groups (*p* ≥ 0.05).

In the ZOI assay, all tested materials were used at a concentration of 300 μg/mL. The inhibition zones were quantitatively measured and expressed in millimeters. The one-sided ZOI width was calculated as follows: ZOI width = (total inhibition zone diameter − paper disc diameter)/2. Data are presented as mean ± SD based on at least three independent measurements.

MIC/MBC analysis was performed using two-fold serial dilutions with final concentrations ranging from 25.00 to 0 μg/mL. The MIC values of Ag-TCPP, Cu-TCPP, and Ce-TCPP against S. mutans were 3.13, 12.50, and 12.50 μg/mL, respectively. These results indicate that the Ag-TCPP/Ag-based composite achieved bacterial growth inhibition at a lower concentration than the Cu- and Ce-containing materials.

Morphological examination by scanning electron microscopy (SEM) (Figure 3D) provided further evidence of the antibacterial mechanism, revealing severe cell envelope damage and leakage of intracellular contents in *S. mutans* after exposure to the MOF formulations. Confocal laser scanning microscopy (CLSM) analysis (Figure 3E) provided a visual confirmation of bactericidal effects, demonstrating near-total eradication of *S. mutans* with Ag-TCPP treatment, substantial bacterial death with Cu-TCPP, and relatively low efficacy with Ce-TCPP. The visual findings were confirmed by quantitative assessment of PI/SYTO9 fluorescence ratios (Figure 3F), demonstrating a dose-dependent antibacterial activity of the three MOFs, with Ag-TCPP exhibiting superior performance.

**Figure 4:**
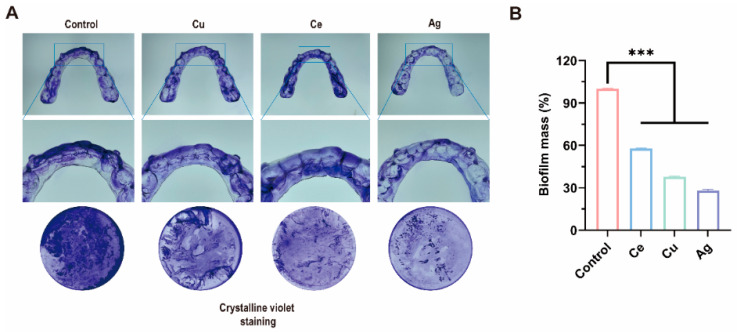
Anti-biofilm formation: (**A**) Representative images of crystal violet-stained *Streptococcus mutans* (*S. mutans*) biofilms formed on uncoated (Control) and MOF-coated aligners (Ag-TCPP, Cu-TCPP, and Ce-TCPP). (**B**) Quantitative analysis of biofilm biomass by crystal violet assay (absorbance at 570 nm), ***: *p* < 0.001.

Biofilm inhibition assays, measured through crystal violet staining (570 nm absorbance) and subsequent statistical analysis, revealed significantly greater biofilm suppression by Ag-TCPP compared to its counterparts (Figure 4A,B).

### 2.5. Biocompatibility Assessment of MOF-Coated Aligners

**Figure 5:**
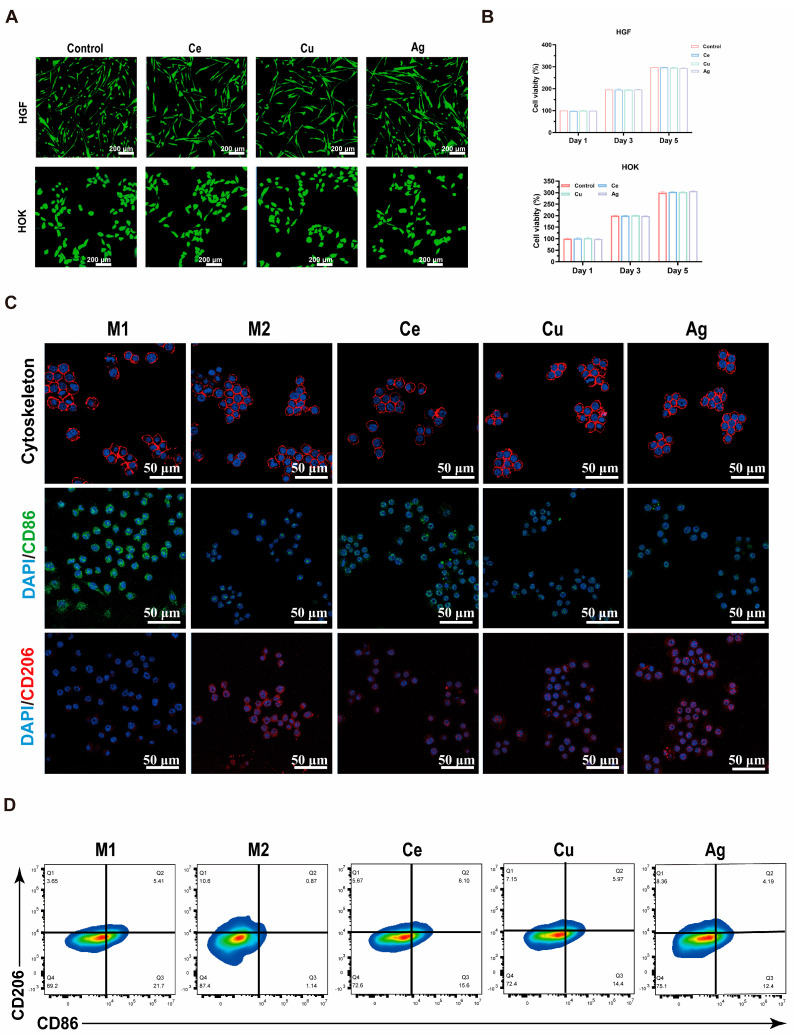
Biocompatibility and immunomodulatory potential: (**A**) Live/Dead staining of cells treated with three TCPP-coated orthodontic appliances. (**B**) Cell proliferation assay of extracts from three types of MOF-coated orthodontic appliances. (**C**) Immunofluorescence staining of RAW 264.7 macrophages for M1 (CD86) and M2 (CD206) markers after treatment with MOF extracts. (**D**) Flow cytometric analysis of macrophage polarization phenotypes (M1/M2) in RAW 264.7 cells exposed to the three TCPP MOFs.

The biocompatibility of the MOF-functionalized aligners was systematically assessed using CCK-8 assays in HOK and HGF. CSLM of Live/Dead-stained cells (Figure 5A) revealed normal cell morphology and well-organized cytoskeletal structures, with no observable signs of membrane compromise, apoptosis, or cellular contraction, confirming the high cytocompatibility of the MOF--modified surfaces. Consistent with these findings, comparative analysis showed no statistically significant reduction in cell viability relative to the control groups (*p* > 0.05), suggesting negligible cytotoxicity of the coatings (Figure 5B).

### 2.6. Macrophage Polarization and Flow Cytometric Analysis of TCPP MOFs

The three TCPP-based metal organic frameworks (MOFs) facilitated macrophage polarization from the pro-inflammatory M1 phenotype to the anti-inflammatory M2 phenotype, as demonstrated by the immunofluorescence analysis (Figure 5C). Fluorescence-based quantification revealed a pronounced downregulation of the pro-inflammatory marker CD86, alongside a substantial upregulation of the anti-inflammatory marker CD206, consistently observed in all treated samples. The most substantial polarization shift was observed in the case of Ag-TCPP, followed by Cu-TCPP and Ce-TCPP.

Flow cytometry was further performed to quantitatively evaluate the effects of different TCPP−-based materials on RAW264.7 macrophage polarization. Flow cytometric analysis (Figure 5D) confirmed a statistically significant decline in M1 macrophage populations and a dose-dependent increase in M2 populations in all MOF-treated groups. In the representative flow cytometry plots, the proportion of CD86^+^CD206^−^ cells decreased from 21.7% in the M1 control group to 14.4%, 15.6%, and 12.4% in the Ce-TCPP, Cu-TCPP, and Ag-TCPP groups, respectively. In parallel, the proportion of CD86^−^CD206^+^ cells increased from 3.65% in the M1 control group to 7.15%, 5.67%, and 8.36% after treatment with Ce-TCPP, Cu-TCPP, and Ag-TCPP, respectively. Among these groups, Ag-TCPP exhibited the lowest proportion of M1-related cells and the highest proportion of M2-related cells, indicating a stronger tendency to promote M2-like macrophage polarization. Ag-TCPP MOF induced the greatest degree of macrophage phenotype modulation.

## 3. Discussion

This work presents a novel strategy for the surface functionalization of clear aligners using TCPP-based MOFs, providing an innovative solution to the longstanding problem of biofilm-associated complications in orthodontics. While all three MOF coatings (Ag-, Cu-, and Ce-TCPP) exhibited desirable biological and physical properties, the Ag-TCPP coating showed the most favorable overall performance among the tested groups, combining stronger antibacterial and antibiofilm activity with good cytocompatibility while maintaining the mechanical integrity and optical transparency of the aligners.

TCPP-based MOFs have been shown to exhibit robust antibacterial activity, chemical stability, and thermal stability [10]. In this study, coatings for clear aligners were developed using TCPP-based MOFs, and their functional mechanisms were systematically examined. The nature of the metal centers played a pivotal role in directing both the crystal growth patterns and the resulting MOF morphologies [11,12]. Cu-TCPP MOFs exhibited a uniform flocculent structure, in contrast to the bulk-like Ce-TCPP MOFs, whose dense morphology likely arises from the high coordination number and redox activity of Ce^3+^/Ce^4+^ promoting directional crystal growth [13,14]. Ce-TCPP showed relatively weak and broad PXRD diffraction signals, together with relatively low Ce loading, indicating lower crystallinity and/or lower synthesis efficiency. This may partly explain the weaker antibacterial and biological performance of Ce-TCPP compared with Ag-TCPP and Cu-TCPP. Ag-TCPP MOFs, however, formed discrete blocky particles, distinct from the other two types. Previous studies have shown that this phenomenon may be attributed to the relatively weak coordination ability and reducibility of Ag^+^, which promotes accelerated crystal growth rather than nucleation, leading to the development of large, discrete particles and the formation of multiple silver oxide-like species [15,16,17]. FTIR spectroscopy indicated differing levels of -OH participation in coordination, influencing the vibrational signatures [18]. The formation of Ag-TCPP MOFs relied predominantly on Ag–N coordination, with minimal involvement of –OH groups. This variation in bonding mode significantly influences the frameworks’ structural integrity, electronic characteristics, and overall functional performance [5]. The crystal structure and phase composition of the materials, determined by XRD patterns, show that the characteristic diffraction peaks of the Ag-TCPP MOFs sample largely overlap with those of metallic Ag, suggesting that Ag likely exists in a metallic state or a similar structure [19]. Because Ag can coordinate with nitrogen atoms in the porphyrin macrocycle, Ag–N interactions alone cannot fully exclude the formation of metalloporphyrin coordination species. Thus, although the current FT-IR and PXRD results support the presence of Ag–TCPP coordination interactions, they are not sufficient to unambiguously confirm a highly crystalline single-phase Ag-TCPP MOF structure. Therefore, Ag-TCPP is described here as an Ag–TCPP coordination/MOF-based composite.

In the sustained-release test, the Cu and Ce MOFs exhibited a more sustained release of metal ions. However, the Ag group achieved effective antibacterial activity even at a lower concentration. By integrating data on surface metal ion concentrations and release kinetics, a more comprehensive understanding of the antibacterial performance and potential benefits of these three MOF-coated aligners for orthodontic applications can be achieved [20].

The clinical effectiveness of transparent aligners depends heavily on both their mechanical robustness and aesthetic quality. Our study demonstrates that MOF coatings did not compromise the mechanical properties of the Biolon substrate, with measured values remaining comparable to those of the uncoated control. This retention of performance is likely due to the strong interfacial interactions between the MOF layer and the aligner material, which preserve the polymer’s intrinsic molecular structure and mechanical integrity [21,22]. During stretching and tearing processes, the MOF coating and the substrate acted in concert to maintain stability. Moreover, the inherent structural features of the MOFs enabled their incorporation without significantly altering the overall mechanical performance. These findings reassure clinicians that the orthodontic force system remains consistent with these novel aligners, alleviating concerns that force instability could compromise treatment outcomes. In terms of optical properties, all three TCPP-MOF-coated aligners exhibited light transmittance comparable to the uncoated Biolon substrate, reflecting minimal impact of the coatings on transparency. A minor increase in haze was observed in the Ag-TCPP group, which has been attributed in previous studies to its distinctive microstructure formed during coating, promoting enhanced internal light scattering [23]. However, the other two groups maintained their aesthetic performance after modification, preserving the transparent and aesthetic appearance essential for clear aligner therapy [24]. This feature promotes patient acceptance and compliance, particularly among adolescents and adults concerned with aesthetics. The comparable water contact angle values across groups suggest similar surface microstructures between the MOF coatings and the aligner material, contributing to consistent hydrophilicity [25]. The Ag-TCPP coating showed significantly increased surface roughness, which can be attributed to surface defects arising from nanoparticle aggregation during fabrication [26,27]. Further optimization of Ag-MOF deposition protocols is warranted to enhance coating homogeneity and improve patient comfort.

The findings of this study highlight the pronounced antibacterial and anti-biofilm activity of all three MOF-coated aligners, following the potency trend Ag-TCPP > Cu-TCPP ≈ Ce-TCPP. In ZOI assays, Cu-TCPP and Ce-TCPP demonstrated comparatively smaller inhibition zones, whereas Ag-TCPP exhibited a significantly larger zone. This enhanced efficacy of Ag-TCPP is likely due to the sustained and controlled release of metal ions facilitated by metal–ligand coordination [17]. Due to the unique coordination capability of Ag^+^, a composite structure consisting of the MOF and in situ-generated silver nanoparticles (AgNPs) was formed. The synergistic effect between these components thus contributed to the enhanced antibacterial performance. The ZOI assay showed that free TCPP produced a relatively clear inhibition zone, which may be partly related to its favorable diffusion ability in the agar medium. Because ZOI is a diffusion-dependent assay, materials with higher solubility and mobility may generate larger visible inhibition zones. Free TCPP, as an unassembled ligand, may diffuse more readily into the agar. In contrast, after coordination/self-assembly with metal centers, the active components in Ag-TCPP, Cu-TCPP, and Ce-TCPP were incorporated into coordination frameworks or composite structures, leading to slower and more sustained release. Therefore, their antibacterial effects may not be fully reflected by the ZOI width within the limited incubation period. In particular, the Ag-TCPP may exert antibacterial activity through both Ag-related species release and contact-mediated killing, which explains why its ZOI width was comparable to that of TCPP despite its stronger overall antibacterial and antibiofilm performance in other assays. The superior antibacterial performance of the Ag-TCPP should therefore be interpreted based on the combined evidence from MIC/MBC, SEM, Live/Dead staining, and antibiofilm assays. Furthermore, this trend aligned with the metal ion release profiles observed in the sustained-release tests, where the concentrations of Ag^+^, Cu^2+^, and Ce^3+^ ions in artificial saliva surpassed their respective MIC/MBC thresholds. SEM analysis further confirmed extensive disruption of *S. mutans* cells following exposure to MOF extracts, including pronounced membrane rupture and cytoplasmic leakage. The severity of these structural alterations, which varied according to the type and concentration of metal ions [28], closely mirrored their antibacterial potency. These results underscore the pivotal role of metal ions released from the MOF coatings in mediating antibacterial activity. Supporting this, quantitative assessment of PI/SYTO9 staining revealed dose-dependent bactericidal effects, with the Ag-TCPP group exhibiting the highest level of bacterial killing [29]. Crystal violet assays (with absorbance measured at 570 nm) and statistical analysis confirmed the intrinsic association between metal release from MOFs and their associated antimicrobial and biofilm-inhibitory activities [1]. Based on the results of this study, metal-ion release is considered the main experimentally supported antibacterial mechanism. Because no intentional visible-light irradiation was applied during the MIC/MBC assays, the antibacterial results in this study should mainly be interpreted as the intrinsic antibacterial effects of the materials under non-photodynamic conditions. Although TCPP-related ROS generation may potentially contribute to antibacterial activity, this mechanism was not directly verified in the present work by light/dark comparison, ROS detection, or ROS-scavenging experiments. Therefore, further studies involving light/dark antibacterial comparisons, ROS detection, and ROS-scavenging experiments are required to clarify the role of porphyrin-mediated photodynamic effects.

The clinical application of any material demands rigorous evaluation of its biosafety. The biosafety analysis of the three MOF coatings established them as highly biocompatible, exhibiting no cytotoxic effects on HGF and HOK cells. The stable coordination networks of the MOFs likely minimize the non-specific release of ligands or uncontrolled burst release of ions, avoiding adverse effects on mammalian cells. This safety profile, together with the strong antibacterial and anti-inflammatory activity, underscores the therapeutic promise of these coatings. Compared to the control group, all three TCPP-MOF treatments promoted the polarization of macrophages from the M1 to the M2 phenotype. The Ag-TCPP MOFs triggered the most pronounced shift, followed by Cu-TCPP and Ce-TCPP MOFs. The interactions between the released metal ions (Ag^+^, Cu^2+^, and Ce^3+^) and macrophage surface receptors or intracellular signaling pathways are presumably responsible for this immunomodulatory effect, which subsequently modulates gene expression and protein synthesis [30]. Previous studies have shown that when macrophages grow on Ti and TiO_2_ nanotube surfaces, they tend to take on a rounder shape, which is a sign of M1 phenotype polarization. On Ag@TiO_2_-NTs, however, the cells became more elongated, suggesting that Ag helps push macrophages toward the M2 phenotype [31]. It has also been reported that AgNPs can trigger RAW 264.7 macrophages to release IL-8 and activate TNF-α, giving the cells a stronger immune punch [32]. AgNPs also seem to work through the NF-κB pathway, ramping up the expression of downstream inflammatory factors [33]. Interestingly, Ag^+^ appears to do the opposite—it can block the phosphorylation and nuclear translocation of NF-κB p65 in macrophages, which dials down the production of pro-inflammatory mediators. A similar story plays out with Cu^2+^ and Ce^3+^: both ions can put the brakes on NF-κB signaling by stopping IκBα from breaking down and keeping p65 out of the nucleus [34,35]. This leads to lower expression of M1-related genes and a boost in M2 markers like CD206, Arg-1, and IL-10.

That said, copper is a bit of a double-edged sword. Some studies point out that too much copper can actually light up multiple inflammatory pathways—NF-κB, MAPKs, JAK-STAT, and even the NLRP3 inflammasome—so it can push inflammation both ways, pro- and anti-inflammatory outcomes [36]. In our case, the Cu-TCPP MOFs coating released copper ions slowly and at low concentrations, which helped us sidestep cytotoxicity and avoid stirring up too much inflammation. At the same time, it seemed to gently nudge the signaling machinery toward M2 activation, helping keep things balanced and tissues happy. The extent of macrophage polarization induced by the three TCPP MOFs was further quantified by flow cytometry, corroborating the immunofluorescence staining data. Our research group will continue to focus on the mechanistic exploration and translational application of porphyrin-based MOF materials in oral biomedical fields. In future studies, we will further verify the regulatory roles of MOFs in MAPK signaling pathways at the molecular level using Western blot, qPCR, and so on.

While excellent material properties have been demonstrated in this study, certain limitations should be acknowledged. The antibacterial evaluation was performed using a single-species model of *S. mutans*, which, although relevant, does not fully capture the complexity of the polymicrobial oral environment encountered clinically. Furthermore, the long-term stability of the coatings under simulated clinical conditions, including aging, wear resistance, and performance within a complex oral microbiome, remains to be investigated. Future studies will focus on assessing the in vivo biological performance of these MOF coatings in animal models to further validate their biosafety and efficacy. The potential application of the synthesized MOFs for drug delivery will be explored to identify new strategies for enhancing their clinical utility.

Because the exact crystal structures, phase compositions, and metal-to-ligand ratios were not fully resolved in the present study, further structural characterization, including single-crystal XRD, Rietveld refinement, elemental analysis, ICP analysis, and quantitative XPS analysis, is required to determine the definitive chemical formulas of these TCPP-based metal-containing materials.

In addition, the use of DMF/ethanol during coating fabrication represents an important consideration for future translation. Although the treatment was performed under controlled conditions and did not markedly compromise the basic mechanical or optical properties of PETG aligners in this study, residual DMF detection was not performed. Moreover, the long-term stability of the coating under clinically relevant conditions remains to be further validated. Future studies should include residual solvent analysis, long-term artificial saliva immersion, brushing or friction resistance tests, thermocycling, and simulated wearing experiments to further evaluate the safety, durability, and long-term stability of the modified aligners. Milder surface activation strategies or DMF-free coating methods should also be explored to improve the translational potential of this coating approach.

Post-antibacterial or post-leaching PXRD characterization was not performed in the present study. Therefore, although the coatings showed antibacterial activity under the tested conditions, their long-term structural stability and degradation behavior require further investigation. Future studies will include post-test PXRD analysis, long-term immersion tests, and additional structural characterization to clarify the stability of these metal–TCPP coordination/MOF-based coatings.

Although AgCl or other poorly soluble silver salts may also provide sustained Ag^+^ release, the purpose of using TCPP in this study was not merely to construct a passive Ag^+^-releasing reservoir. TCPP was selected as a multifunctional coordination-based coating platform because its four peripheral carboxyl groups can provide multiple coordination sites for metal ions and facilitate coating formation. In addition, the porphyrin macrocycle provides a tunable molecular framework for constructing metal–organic functional coatings. Compared with simple Ag^+^-releasing salts, the TCPP-based system allows different metal centers, including Ag, Cu, and Ce, to be incorporated and compared within a similar ligand framework. This design is useful for evaluating the effects of different metal ions on antibacterial activity, antibiofilm performance, cytocompatibility, and cellular responses. However, a direct comparison with AgCl-based coatings was not performed in the present study. Therefore, whether the TCPP-based coating provides a more favorable Ag^+^ release profile than AgCl requires further investigation. Future studies will compare TCPP-based coatings with AgCl-based or other poorly soluble silver salt systems in terms of Ag^+^ release behavior, coating stability, antibacterial performance, cytocompatibility, and long-term applicability.

Another limitation is that the detailed crystallographic structures and phase purity of the metal–TCPP materials were not fully determined. Although the FT-IR and PXRD results supported the formation of metal–TCPP coordination structures, further characterization, including PXRD comparison with the free TCPP ligand, comparison with reported or simulated PXRD patterns, and additional spectroscopic analysis, is needed to further confirm the detailed structures. Moreover, because metal ions may coordinate with nitrogen atoms in the porphyrin macrocycle, the formation of metalloporphyrin coordination species cannot be fully excluded based only on the current data. The composite nature of Ag-TCPP, including Ag-TCPP coordination components and metallic Ag secondary phases, may partly contribute to its relatively strong antibacterial activity, increased surface roughness, and increased haze. However, the long-term structural stability and phase evolution of this Ag-containing coating remain to be further investigated. Post-antibacterial or post-leaching PXRD characterization was not performed in the present study. Therefore, although the coatings showed antibacterial activity under the tested conditions, their long-term structural stability and degradation behavior require further investigation. Future studies will include post-test PXRD analysis, long-term immersion tests, and additional structural characterization to clarify the stability of these metal–TCPP coordination/MOF-based coatings.

## 4. Materials and Methods

### 4.1. Materials

Biolon aligner sheets were procured from Dreve Company (Unna, Germany). Cerium nitrate and meso-tetra(4-carboxyphenyl)porphyrin, also referred to as H_4_TCPP (C_48_H_30_N_4_O_8_), were obtained from Aladdin Biotechnology Co., Ltd. (Shanghai, China). The Cell Counting Kit-8 (CCK-8) was purchased from Beyotime Biotechnology Co., Ltd. (Shanghai, China). Brain heart infusion (BHI) broth was supplied by Feijing Biotechnology Co., Ltd. (Fuzhou, China). Dulbecco’s Modified Eagle Medium (DMEM), Roswell Park Memorial Institute (RPMI) 1640 medium, fetal bovine serum (FBS), and other cell culture reagents were acquired from Thermo Fisher Scientific (Waltham, MA, USA). Copper nitrate, N,N-dimethylformamide (DMF), polyvinylpyrrolidone K30 (PVP), silver nitrate, benzoic acid, and other chemicals were purchased from Sinopharm Chemical Reagent Co., Ltd. (Shanghai, China). Artificial saliva (neutral, pH 6.8) was obtained from Fuzhou Feijing Biotechnology Co., Ltd. (Fuzhou, China). The artificial saliva was designed for scientific research applications and complied with the ISO 10271 standard. The artificial saliva was used directly in the sustained-release assay without further in-house preparation. Human normal oral keratinocytes (HOK), human gingival fibroblasts (HGF), and mouse mononuclear macrophage cells (RAW264.7) were obtained from Procell Life Science & Technology Co., Ltd. (Wuhan, China). *Streptococcus mutans* (ATCC 25175) was acquired from Huayueyang Biotechnology Co., Ltd. (Beijing, China). All reagents and solvents were of analytical grade and used without further purification.

### 4.2. Synthesis and Characterization of TCPP-Based Metal Coordination Materials

TCPP-based metal coordination/composite materials were synthesized via a coordination-driven self-assembly process. The organic ligand used in this study was H_4_TCPP, with the molecular formula C_48_H_30_N_4_O_8_. Three separate precursor mixtures were prepared as follows: (1) AgNO_3_ (10 mg) was mixed with TCPP (4 mg), PVP K30 (10 mg), and benzoic acid (0.1124 g); (2) Cu(NO_3_)_2_·3H_2_O (10 mg) was mixed with TCPP (4 mg), PVP K30 (10 mg), and benzoic acid (0.1124 g); and (3) Ce(NO_3_)_3_·6H_2_O (10 mg) was mixed with TCPP (4 mg), PVP K30 (10 mg), and benzoic acid (0.1124 g). Each mixture was dissolved in a solvent composed of DMF (15 mL) and anhydrous ethanol (5 mL), followed by ultrasonication for 15 min. The solution was then transferred to a round-bottom flask and heated at 90 °C in an oil bath under gentle stirring at 500 rpm for 3 h [37,38]. After cooling to room temperature, the mixture was ultrasonicated for another 15 min and centrifuged at 2000 rpm for 10 min. The supernatant was discarded, and the precipitate was washed three times with anhydrous ethanol by repeated redispersion and centrifugation. The purified products were stored in anhydrous ethanol at 4 °C for subsequent use.

Benzoic acid was used as a monocarboxylic acid modulator in the synthesis. Because its amount was much higher than that of TCPP, benzoic acid may competitively coordinate with metal ions, thereby regulating the coordination assembly rate between metal ions and the carboxylate groups of TCPP, slowing rapid nucleation, and influencing crystal growth and particle morphology through a capping or modulating effect. PVP K30 was used mainly as a surface stabilizer and dispersant. The carbonyl groups of PVP may weakly interact with metal ions or particle surfaces, thereby reducing particle aggregation and improving the dispersion stability of the reaction system. In addition, PVP may also improve the film-forming behavior of the coating suspension and promote more uniform particle deposition on the aligner surface.

Because definitive crystallographic structures and exact metal-to-ligand ratios were not fully determined in the present study, the obtained products are described using nominal composition expressions rather than fixed crystal chemical formulas. The Cu- and Ce-containing samples are described as Cuₓ(TCPP)y and Ceₓ(TCPP)y, respectively, representing metal–TCPP coordination materials. Because metallic Ag diffraction peaks were observed in the PXRD pattern of the Ag-containing sample, Ag-TCPP is more cautiously described as Agₓ(TCPP)y/Ag^0^, indicating the possible coexistence of Ag-TCPP coordination components and metallic Ag crystalline phases.

The as-synthesized TCPP-based metal coordination/composite materials were analyzed for their morphology and elemental composition using scanning electron microscopy (SEM, Carl Zeiss AG, Oberkochen, Germany) coupled with energy-dispersive X-ray spectroscopy (EDS). Fourier-transform infrared spectroscopy (FTIR, Bruker Corporation, Ettlingen, Germany) and X-ray diffraction (XRD, Rigaku Corporation, Akishima, Japan) were conducted on dried powders to identify characteristic functional groups and evaluate their crystalline features and phase compositions.

### 4.3. Preparation and Characterization of MOF-Coated Aligners

Biolon aligner sheets were rinsed three times with deionized water and dried before coating. The three types of MOFs were dispersed in a 75% DMF/25% ethanol solution by ultrasonication to obtain a homogeneous suspension. In this system, DMF was used to mildly activate the PETG surface and improve the interfacial adhesion of MOF particles, while ethanol helped regulate solvent compatibility and dispersion. To balance effective surface activation with the preservation of the PETG substrate, all procedures were performed at room temperature, and the solvent exposure time was carefully controlled. The MOF suspension was then spin-coated onto the aligner surface at 100 rpm for 30 s. After coating, unbound particles were removed by rinsing with deionized water. The surface morphology of the coated aligners was examined by scanning electron microscopy (SEM) [39].

### 4.4. Sustained-Release Testing of Aligner Coatings

Inductively coupled plasma mass spectrometry (ICP-MS, Thermo Fisher Scientific Inc., Waltham, MA, USA) was employed for the quantitative analysis of initial metal ion concentrations on the MOF-coated aligners with reference to standard calibration curves. To evaluate sustained release kinetics, MOF-coated aligner sheets were incubated in artificial saliva for 1–5 days in polytetrafluoroethylene (PTFE) digestion vessels. Released metal ions were quantified daily via inductively coupled plasma optical emission spectroscopy (ICP-OES, Thermo Fisher Scientific Inc., Waltham, MA, USA). By comparison with standard curves, the cumulative release of MOFs was monitored over a period of five days.

### 4.5. Physical Performance Evaluation of MOF-Coated Aligners

The mechanical properties of uncoated and MOF-coated aligner sheets were systematically analyzed using stress–strain profiling and right-angle tear testing on a universal electronic material testing system (SUNS, Shenzhen Suns Technology Stock Co., Ltd., Shenzhen, China), adhering to the YY/T 1819-2022 standard.

$$Stress=\frac{F}{A0}\;(\mathrm{Unit}:\;\mathrm{MPa})$$

$$Strean=\frac{L1}{L0}\times 100\%\;(\mathrm{Unit}:\;\mathrm{mm})$$

$$Tear\;strength=\frac{Fmax}{d}$$

where F: maximum tear load (recorded in Newtons, N); d: thickness of the specimen at the notch (in millimeters, mm).

Optical performance was evaluated by determining light transmittance and haze in accordance with the GB/T 2410 standard.

$$Transmittance:Tx=\frac{T_{total}}{T_{incident}}\times 100\%$$

$$Haze:H=\frac{T_{scattered}}{T_{total}}\times 100\%$$

where T_total_: total transmitted light flux.

T_incident_: incident light flux.

T_scattered_: scattered light flux (light deviating > 2.5° from the incident beam).

The water contact angle (WCA) of MOF-coated aligners was measured to evaluate the surface wettability of the samples. Both MOF-coated and uncoated aligner sheets were sectioned into 10 mm × 10 mm pieces while maintaining clinically relevant thickness specifications. The sample stage was leveled using a spirit level to ensure the gravitational direction of the droplet was perpendicular to the imaging plane. An ultrapure water droplet (2 μL) was deposited at the center of the sample using a microsyringe, with the needle tip positioned 1–2 mm above the surface. After allowing the droplet to stabilize (no significant spreading within 5 s), side-view images of the droplet were captured using a contact angle goniometer (Zhongchen Digital Technology Equipment Co., Ltd., Shanghai, China).

Surface roughness of the MOF-coated aligners was analyzed to evaluate comfort during wear, staining propensity, and long-term biocompatibility with oral soft tissues. Sample preparation was conducted in accordance with the procedure established for WCA analysis. Using a portable roughness tester (Mahr GmbH, Göttingen Germany), the surface roughness measurements were performed at five distinct regions on individual samples to minimize the influence of scratches and environmental factors.

### 4.6. Antibacterial Evaluation of MOF-Coated Aligner

The antibacterial efficacy of MOF-coated aligners was evaluated against *Streptococcus mutans*: The zone of inhibition (ZOI), minimum inhibitory concentration (MIC), and minimum bactericidal concentration (MBC) were determined for three MOF solutions. were determined for three MOF solutions. Anti-biofilm activity was evaluated using crystal violet staining, bactericidal activity was assessed using MOF-coated aligner extracts, and Live/Dead staining was performed on *S. mutans* treated with MOF-coated aligner extracts.

#### 4.6.1. Zone of Inhibition (ZOI) Assay for Three TCPP MOFs

Bacterial suspensions were diluted to 1 × 10^8^ CFU/mL, and 20 μL of the suspension was spread onto BHI agar plates using the spread plate technique. Sterile paper discs impregnated with test solutions (three MOFs (300 μg/mL) and TCPP (300 μg/mL)) were placed on the inoculated agar surfaces. Plates were incubated at 37 °C for 24 h, and the ZOI around each disc was measured.

The inhibition zone width was calculated as follows:

Inhibition zone width = (total inhibition zone diameter − disc diameter)/2

#### 4.6.2. Determination of Minimum Inhibitory Concentration (MIC) and Minimum Bactericidal Concentration (MBC) of Three MOF Solutions Against *Streptococcus mutans*

The antibacterial activities of Ag-TCPP, Cu-TCPP, and Ce-TCPP MOFs against *Streptococcus mutans* (ATCC 25175) were evaluated using the broth microdilution method according to CLSI guidelines (M07-A10) with modifications. A series of two-fold serial dilutions of the three MOF solutions was prepared in sterile brain heart infusion (BHI) broth, yielding final concentrations ranging from 25.00 to 0 μg/mL. A bacterial inoculum standardized to 1 × 10^5^ CFU/mL, as confirmed by viable colony enumeration, was mixed with equal volumes of MOF solutions in sterile tubes. The mixtures (200 μL per well) were transferred to a sterile 96-well plate (Corning^®^ Costar^®^).

Considering the photoactivity of TCPP under visible-light irradiation, the light exposure conditions during MIC and MBC testing were carefully controlled. No intentional visible-light irradiation was applied during the assays. Except for short-term exposure to dim ambient indoor light during solution preparation, two-fold serial dilution, and bacterial inoculation, all procedures were performed under light-protected conditions as much as possible. After sample loading, the 96-well plates were immediately wrapped with aluminum foil and incubated at 37 °C for 24 h in the dark. Therefore, the MIC and MBC results obtained in this study were measured under non-photodynamic conditions.

Each of the three different samples was subjected to three independent tests to ensure accuracy and reproducibility. The 96-well plate was then incubated at 37 °C for 24 h, following which all wells at a wavelength of 600 nm were recorded using a microplate reader. The minimum inhibitory concentration (MIC) was defined as the lowest concentration of the MOF solution that showed reduced colony counts when compared to the negative control group. The minimum bactericidal concentration (MBC) was defined as the lowest concentration at which no visible bacterial colonies were observed.

#### 4.6.3. Bactericidal Activity of MOF-Coated Aligner Extracts

Freshly prepared *S. mutans* cultures adjusted to 1 × 10^6^ CFU/mL in BHI broth were prepared and stored at 4 °C. Four sterile silicon wafers (G1–G4) were assigned to experimental groups: G1 (control) received 1 mL of 0.9% saline, whereas G2–G4 were treated with 1 mL of extracts from the three MOF-coated aligners. Wafers were co-cultured with bacterial suspensions at 37 °C for 24 h, rinsed with saline, fixed overnight in 2.5% glutaraldehyde, and dehydrated through an ethanol gradient (10%, 30%, 50%, 70%, and 100%). After air-drying at 37 °C, the silicon wafers were sputter-coated with gold prior to scanning electron microscopy (SEM) to assess bacterial morphological changes.

#### 4.6.4. Live/Dead Staining of *S. mutans* Treated with MOF-Coated Aligner Extracts

*S. mutans* colonies were inoculated into BHI broth and incubated at 37 °C for 24 h with orbital shaking at 150 rpm. Bacterial suspensions were diluted in saline to 1 × 10^6^ CFU/mL, and 100 μL aliquots were dispensed into 96-well plates. MOF-coated aligner extracts were sterilized under UV light for 2 h, and 100 μL of each extract was added to the respective wells. Following 4 h of incubation at 37 °C, 10 μL of the mixture was taken from each well, gently combined with 10 μL of SYTO9 stain on a glass slide, and incubated in the dark for 15 min. Propidium iodide (PI) was subsequently added, and the samples were incubated in the dark for another 15 min. Stained cells were imaged using a laser-scanning confocal microscope (Carl Zeiss Microscopy GmbH, Jena, Germany).

#### 4.6.5. Crystal Violet Staining for Anti-Biofilm Activity

MOF-coated and blank aligners were cut into 11 mm diameter disks and placed in the wells of 24-well plates, with untreated aligners serving as controls. The discs were immersed in *S. mutans* suspensions (1 × 10^8^ CFU/mL, 1 mL) for 48 h to allow the formation of biofilm. Following incubation, disks were rinsed with PBS to eliminate non-adherent bacteria, fixed with 4% paraformaldehyde (15 min, room temperature), and stained with 0.1% crystal violet (1 mL/well) for 1 h. Excess stain was removed by washing three times with PBS. The stained biofilms were imaged, solubilized in 30% acetic acid (1 mL/well), and quantified by measuring absorbance at 570 nm using a microplate reader. For clinically relevant evaluation, MOF-coated and uncoated aligner sheets were thermoformed by pressure molding, trimmed to the final aligner geometry, and subjected to the same crystal violet staining protocol. Biofilm density was compared among four experimental groups.

### 4.7. Biocompatibility of MOF-Coated Aligners

Human gingival fibroblast (HGF) and human oral keratinocyte (HOK) cultures in the exponential phase were seeded into 96-well plates at a density of 1 × 10^3^ cells/well and allowed to incubate for 24 h. The cells were subsequently co-cultured with extracts from three types of MOF-coated aligners for 1, 3, and 5 days, and absorbance was measured at 450 nm using a microplate reader (BioTek Instruments, Inc., Winooski, VT, USA). For Live/Dead staining, HGF and HOK cells (3 × 10^4^ cells per confocal dish) were exposed to aligner extracts and incubated in serum-free medium containing a mixture of Calcein-AM and propidium iodide (PI). Using a confocal microscope (Leica Microsystems Trading Co., Ltd., Shanghai, China), live cells (green fluorescence) and dead cells (red fluorescence) were visualized.

### 4.8. Evaluation of Anti-Inflammatory Properties of MOF-Coated Aligners

MOF-coated aligners were assessed for their anti-inflammatory characteristics through immunofluorescence staining and flow cytometry to examine the polarization of RAW 264.7 macrophages. For this purpose, RAW 264.7 cells were cultured under standard conditions (37 °C, 5% CO_2_) and seeded onto sterile coverslips (12 mm diameter) in 24-well plates at a density of 2 × 10^4^ cells per well. Extracts derived from the three MOF-coated aligners were applied to the wells. For cytoskeletal visualization, cells mounted on coverslips were stained with phalloidin to label actin filaments and incubated in the dark at room temperature for 45 min. Following three PBS washes to remove unbound stain, the coverslips were mounted onto glass slides and imaged using a confocal microscope. For antibody staining, cells were blocked with 1% Bovine Serum Albumin (BSA) for 1 h and incubated with fluorescent secondary antibodies (1:500–1:1000) in the dark at room temperature for 1 h. After PBS washes, nuclei were counterstained with 1 μg/mL 4′,6-diamidino-2-phenylindole (DAPI) for 5 min in the dark, followed by three additional PBS washes. Samples were then imaged using a laser-scanning confocal microscope.

RAW 264.7 cells were polarized into M1 and M2 phenotypes. For flow cytometric analysis, CD86 and CD206 were used as representative markers for M1- and M2-related macrophage phenotypes, respectively. CD86^+^CD206^−^ cells were defined as M1-related cells, whereas CD86^−^CD206^+^ cells were defined as M2-related cells. The proportions of these cell populations were quantified from representative flow cytometry plots. Cell suspensions were co-stained with anti-CD86 and anti-CD206 antibodies at 4 °C in the dark for 30 min. After PBS washes to remove unbound antibodies, cells were analyzed by flow cytometry.

### 4.9. Statistical Analysis

Data derived from independent experiments are presented as mean ± standard deviation (SD). Multiple comparisons were conducted using one-way analysis of variance (ANOVA). All statistical evaluations were performed using SPSS 26.0, with each measurement conducted in triplicate. Significance levels are denoted as follows: *: *p* < 0.05, **: *p* < 0.01, ***: *p* < 0.001, ****: *p* < 0.0001, ns: no significance.

## 5. Conclusions

This work successfully synthesized and characterized three distinct metal–organic frameworks (MOFs), Ag-TCPP, Cu-TCPP, and Ce-TCPP, which were subsequently employed to fabricate MOF-coated clear orthodontic aligners. Of the MOF-coated aligners evaluated, those incorporating Ag-TCPP demonstrated superior antibacterial and anti-biofilm efficacy, maintained high biocompatibility, and elicited enhanced immunomodulatory responses under inflammatory conditions relative to Cu-TCPP and Ce-TCPP coatings. These findings highlight the potential of TCPP-MOF-coated aligners for clinical orthodontic applications, supporting the advancement of invisible orthodontic technology and offering improved treatment options. Moreover, this work provides valuable guidance for the development of other novel materials for oral medical devices.

## Data Availability

The original contributions presented in this study are included in the article/Supplementary Material. Further inquiries can be directed to the corresponding authors.

## Supplementary Materials

The following supporting information can be downloaded at: https://www.mdpi.com/article/10.3390/ijms27125411/s1.
