# Supplementary figures and images for: Enhanced Antibacterial and Immunomodulatory Porphyrin-Based MOF Coatings for PETG Clear Aligners: A Comparative Study of Ag, Cu, and Ce Metal Centers
Source: Int J Mol Sci. 2026 Jun 16;27(12):5411. doi: 10.3390/ijms27125411 (PMC13300317; doi:10.3390/ijms27125411)

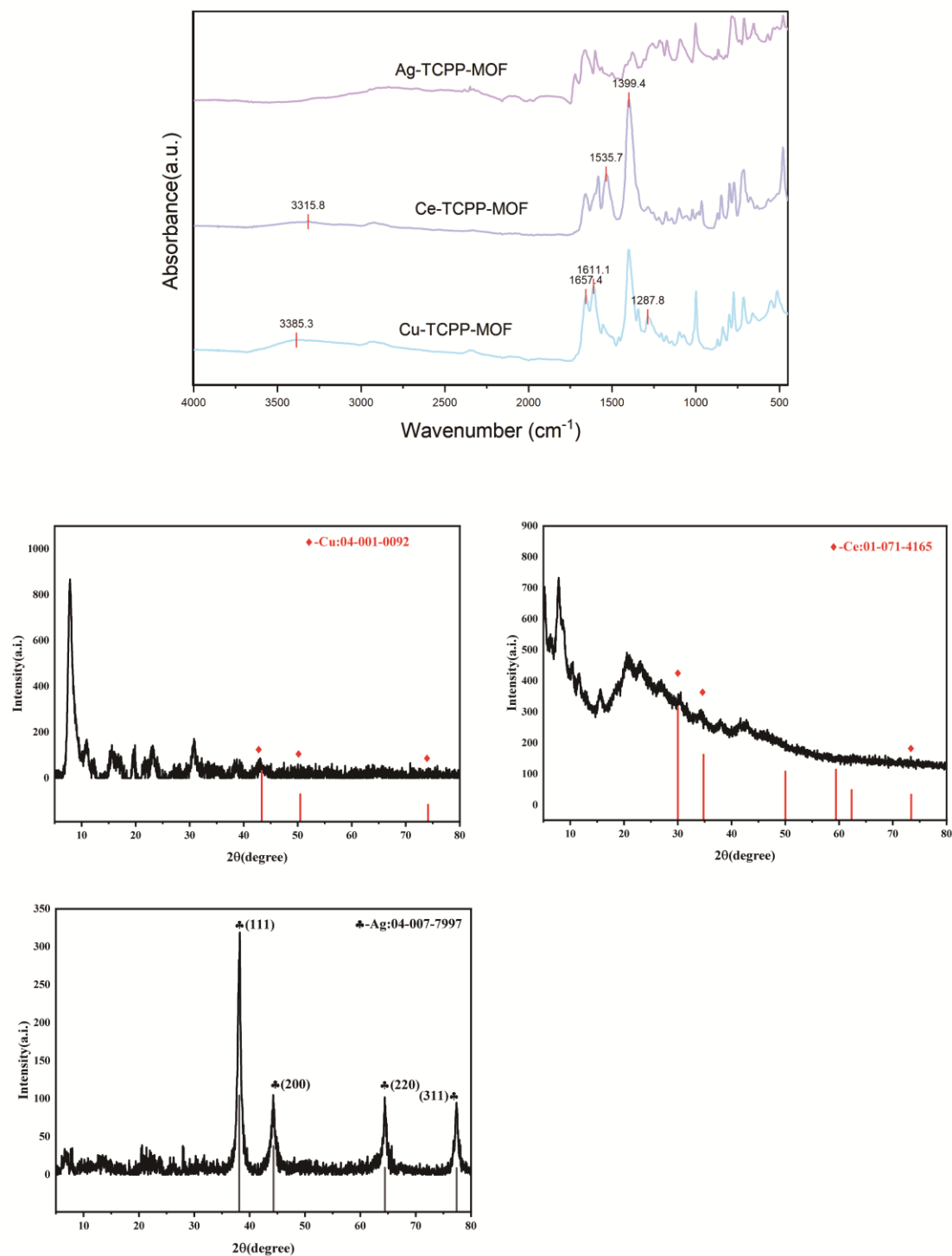

Figure S1.

Enlarged FT-IR spectra and PXRD patterns of Ag-TCPP, Cu-TCPP, and Ce-TCPP materials.

Supplement: Supplementary file 1 [file ijms-27-05411-s001.zip › ijms-4289171-supplementary.pdf]
